
JOURNAL INFORMATION
==============================
NLM Title Abbreviation: JBRA Assist Reprod
Journal ID: JBRA Assist Reprod
NLM Journal ID: 101684552
Journal ID: jbra

JBRA Assisted Reproduction

ISSN: 1517-5693
EISSN: 1518-0557
Publisher: Brazilian Society of Assisted Reproduction

ARTICLE INFORMATION
==============================
PMCID: PMC7558887
PMID: 33021751
DOI: 10.5935/1518-0557.20200072
Article version: 1
Subjects: Oral Presentations

Abstracts of the 24th Annual Congress of the SBRA, 2020 (Virtual Meeting)

Publication date: 2020 Oct-Dec
Print publication date: 2020 Oct-Dec
Volume: 24
Issue: 4
First page: 518
Last page: 524
License: This is an Open Access article distributed under the terms of the Creative Commons Attribution License, which permits unrestricted use, distribution, and reproduction in any medium, provided the original work is properly cited.
License URL: https://creativecommons.org/licenses/by/4.0/

==============================


O-01. Trisomy causes accelerated embryonic development. Is that an indication for preimplantation genetic test for aneuploidy in in vitro fertilized embryos?

Gomes A.P. 1
Martin H. de 1 2
Fujii M.G. 1
Conatti M. 1
Bonetti T.C.S. 3
Monteleone P.A. 1 2
1 Centro de Reprodução Humana Monteleone, São Paulo. Brazil
2 Disciplina de Ginecologia - Departamento de Obstetrícia e Ginecologia. Faculdade de Medicina da Universidade de São Paulo (FMUSP), São Paulo. Brasil
3 Departamento de Ginecologia. Escola Paulista de Medicina da Universidade Federal de São Paulo (EPM-UNIFESP), São Paulo. Brazil

O-02. Motherhood plan: has it changed in face of the COVID-19 pandemics?

Braga D.P.A.F. 1 2
Setti A.S. 1 2
Iaconelli Jr. A. 1 2
Borges Jr. Edson 1 2
1 Fertility Medical Group - São Paulo/SP
2 Instituto Sapientiae - Centro de Estudos e Pesquisa em Reprodução Assistida- São Paulo/SP

O-03. Can we predict aneuploidy with morphokinetics parameters?

Nicolielo M. 1
Jacobs C. 1
Erberelli R. 1
Mendez F. 1
Belo A. 1
Fanelli M. 1
Aiello B. 1
Motta E.L.A. 2
Lorenzon A.R. 3
1 Huntington Medicina Reprodutiva, Embryology, São Paulo, Brazil.
2 Huntington Medicina Reprodutiva/Federal University of São Paulo, Clinical Director /Associate Professor, São Paulo, Brazil.
3 Huntington Medicina Reprodutiva, Research & Development, São Paulo, Brazil.

O-04. Early and late paternal contribution to cell division of embryos in a time-lapse incubation system

Setti A.S. 1 2
Braga D.P.A.F 1 2
Provenza R.R. 1
Iaconelli Jr. A. 1 2
Borges Jr. E. 1 2
1 Fertility Medical Group - São Paulo/SP
2 Instituto Sapientiae - Centro de Estudos e Pesquisa em Reprodução Assistida - São Paulo/SP

O-05. Oocyte ability to repair sperm DNA fragmentation: The effect of maternal age on ICSI outcomes

Borges Jr. E. 1 2
Braga D.P.A.F. 1 2
Setti A.S. 1 2
Provenza R.R. 1
Iaconelli Jr. A. 1 2
1 Fertility Medical Group - São Paulo/SP
2 Instituto Sapientiae - Centro de Estudos e Pesquisa em Reprodução Assistida - São Paulo/SP

O-06. Zika virus infectivity in human ovarian granulosa cells

Araújo L.B. 1
Bloise F.F. 1
Souza M.C.B. 2
Antunes R.A. 2
Mancebo A.C.A. 2
Coelho S.V.A. 3
Arruda L.B. 3
Ortiga-Carvalho T.M. 1
1 Institute of Biophysics Carlos Chagas Filho, Universidade Federal do Rio de Janeiro (UFRJ), Rio de Janeiro, RJ, Brazil.
2 Fertipraxis Centro de Reprodução Humana, Centro Médico Barrahopping - Rio de Janeiro, RJ, Brazil.
3 Institute of Microbiology Paulo de Góes, Universidade Federal do Rio de Janeiro (UFRJ), subsolo - Rio de Janeiro, RJ, Brazil.

O-07. Validation process of free-DNA medium collection for noninvasive preimplantation genetics test for aneuploidy (niPGT-A)

Petersen C.G. 1 2
Vagnini L. 1
Renzi A. 1
Oliveira J.B.A. 1 2
Dieamant F. 2
Petersen B. 2
Canas M.C.T. 1
Oliani A.H. 3
Nakano R. 4
Almodin C.G. 5
Marcondes C. 6
Ceschin A. 7
Amaral A. 8
Borges Jr E. 9
Castelo Branco A. 10
Soares J.B. 11
Lopes J. 12
Franco Jr. J.G. 1 2
1 Paulista Center for Diagnosis-Research and Training, Ribeirao Preto, Brazil.
2 Centre for Human Reproduction Prof. Franco Jr, Ribeirão Preto, Brazil.
3 São Jose do Rio Preto School of Medicine FAMERP, São Jose do Rio Preto, Brazil.
4 Ferticlin Human Fertility Clinic, Research, São Paulo, Brazil.
5 Materbaby, Maringa, Brazil.
6 Santista Nucleus of Human Reproduction, Santos,Brazil.
7 Feliccita Fertility Institute, Curitiba, Brazil.
8 Genesis Human Reproduction Assistance Center, Brasilia, Brazil.
9 Fertility Medical Group, Research, São Paulo, Brazil.
10 Art Fertil Human Reproduction Clinic, Recife,Brazil.
11 Alpha Project - Alliance of Assisted Fertilization Laboratories, São Paulo, Brazil.
12 CENAFERT, Salvador, Brazil.

O-08. Surrogacy: the main indications and couple’s biggest challenges, an 18-year psychology experience in a reproductive medicine clinic in Brazil.

Avelar CC 1
Leite FTF 1
Pereira MR 1
Marinho RM 1
Caetano JPJ 1
1 Huntington/Pró-criar Medicina Reprodutiva

O-09. Three-dimensional (3D) in vitro culture using magnetic levitation system of bovine ovary fragments maintain tissue viability and balanced oxidative status

Antonino D.C. 1
Soares M.M. 2
Silveira I.N. 1
Watanabe L.G.V. 1
Verzemiassi I.S.B.F. 3
Beletti M.E. 4
Alves K.A. 5
Alves B.G. 6
Rosa-Silva A.C.J.S.
1 Gynecology and Obstetrics Program at Ribeirão Preto Medical School, University of São Paulo.
2 Veterinary Sciences Program at the University of Uberlândia.
3 Sapientiae Institute - Center for Study and Research
4 Institute of Biomedical Sciences, University of Uberlândia.
5 Centro Universitário do Triângulo - UNITRI, Uberlândia.
6 Veterinary, no institucional affiliation.
7 Ribeirão Preto Medical School, University of São Paulo.

O-10. Granulosa cell signaling based bioassay in vitro for personalized stimulation in ART

Paradiso E. 1
Lazzaretti C. 1
Sperduti S. 1
Riccetti L. 1
Nicoli A. 2
Villani M.T. 2
Daolio J. 2
Lispi M. 3
Gravotta E. 3
Orlando G. 3
Simoni M. 1
Casarini L. 1
1 University of Modena and Reggio Emilia, Unit of Endocrinology-Dept. Biomedical-Metabolic and Neural Sciences, Modena, Italy Metabolic and Neural Sciences, Modena, Italy
2 Arcispedale Santa Maria Nuova, Department of Obstetrics and Gynaecology-Fertility center, Reggio Emilia, Italy
3 Merck KGaA, Merck Serono S.p.A - Rome- Italy, Darmstadt, Germany

Abstract presented (Oral Presentation O-282) at the 35th Annual Meeting of the ESHRE, Vienna, Austria 24 to 26 June 2019. Published by permission of Oxford University Press on behalf of ESHRE Oxford University Press License Number: 4851800745400

